# The Football Association Injury and Illness Surveillance Study: The Incidence, Burden, and Severity of Injuries in English Women's Domestic Football—A 5‐Year Prospective Cohort Study

**DOI:** 10.1111/sms.70094

**Published:** 2025-06-27

**Authors:** Bradley Sprouse, Philip Hennis, Pippa Bennett, Steve Kemp, Charlotte Cowie, Abosede Ajayi, Ryan King, Molly Johnson, John Morris, Simon Cooper, Avinash Chandran, Ian Varley

**Affiliations:** ^1^ SHAPE Research Centre, School of Science and Technology Nottingham Trent University Nottingham UK; ^2^ UK Sports Institute Manchester UK; ^3^ The Football Association Burton‐Upon‐Trent UK; ^4^ Women's Professional Leagues Ltd London UK; ^5^ Datalys Center for Sports Injury Research and Prevention Indianapolis Indiana USA

**Keywords:** injury burden, injury incidence, injury surveillance, women's football

## Abstract

The epidemiology of injuries in English women's professional football is yet to be investigated. Therefore, the aim was to examine the incidence, severity, and burden of injury in English women's domestic football. Time‐loss injuries, and match and training exposure, were collected by club medical staff across 5 seasons (2018–2023) from English Women's Super League and Championship teams (93 team seasons). The incidence, severity, burden, cause, onset and patterns of injury were examined. A bootstrapped negative binomial approach was employed to calculate incidence and burden estimates, time‐trend analysis and injury rate ratios (IRR), all with accompanying 95% CI. 2167 injuries were reported, resulting in 76 687 days absent. Of these, 981 match injuries (eliciting 37 269 days absent) and 1186 training injuries (eliciting 39 418 days absent) were reported. Injury incidence was greater in matches than training (20.5 [16.9–24.1] vs. 2.9 [2.0–4.0] injuries per 1000 h [95% CI]; IRR: 7.3, 95% CI: 5.1–10.1). Match injury burden (775.1 days absent/1000 h [95% CI; 630.0–934.9]) was greater than training (96.9 days absent/1000 h [95% CI: 66.1–138.5]) (IRR: 8.3, 95% CI: 5.4–12.0). Training injury incidence decreased by 28% across the study period (*p* = 0.041), with no significant seasonal changes for match injury incidence (*p* = 0.218) or injury burden (*p* > 0.05). The most common diagnosis was hamstring injury (match: 2.37; training: 0.38; injuries/1000 h), while ACL injuries were the most burdensome (match: 136.31; training: 10.86 days absent/1000 h), with no significant seasonal changes observed (*p* > 0.05). Overall, the present study showed that injury incidence and burden were greater in matches than training, with incidence decreasing by 28% across the study period in training in English women's domestic football. Hamstring injuries were the most common injury diagnosis, and ACL injuries were the most burdensome; illustrating the need to continue to develop prevention strategies for these injuries. The present study provides injury surveillance information in English women's domestic football and can be a benchmark for the success of future injury prevention strategies.

## 1 Introduction

In England, the Football Association (FA) Women's Super League began in 2011 and changed the landscape of elite women's football. This key structural alteration has been associated with growth in funding, supporter attendance and the number of elite players and support staff in professional women's football in England [1]. These off‐field improvements have been accompanied by substantial on‐field developments, with modern professional women's football globally becoming more technical, tactical, and physically demanding [2, 3]. These wide‐ranging modifications to the characteristics of the professional women's game may well have influenced injury occurrence given the associations between high exposure, load and injury risk [4, 5]. However, studies estimating injury incidence largely pre‐date the modern, professional era of Women's domestic (league‐based) football (1988–2015 [6]) and it is unclear whether those studies represent the injury occurrence and specific injury characteristics in the modern game.

A recent review of studies associated with injury in women's football published in 2021 [6] shows that, since 1991 [7], only a small number of research studies have sought to describe and understand the complete landscape of injuries incorporating incidence, severity, mechanisms, type, and location in the elite women's game [6]. These studies show the incidence of injury to be between 1.9 (Club Football, USA) and 8.4 (Club Football, Netherlands) injuries/1000 h for all football related activity, and those occurring specifically during matches and training to range between 12.6 (Club Football, USA) and 30.3 (Club Football, Netherlands) injuries/1000 h, and 1.2 (Club Football, USA) and 7.0 (Club Football, Sweden) injuries/1000 h, respectively [6]. Recently, the UEFA Women's Elite Club Injury Study (WECIS, 2018–2022) reported a match and training injury incidence of 18.4 and 4.8 injuries/1000 h [8], which lay within the ranges identified in the review.

A characteristic of the studies that have been published is the variability of the estimates reported. This variability may stem from the utilization of different methodological approaches to the definition and reporting of injury. However, the key issue is that the estimates are typically derived from relatively small data sets that are collected during a single season [7, 9, 10, 11, 12, 13, 14, 15], or for a single team [16, 17]. An additional factor that likely impacts injury occurrence in women's football is the level of competition [6], which will differ across and within studies and has changed substantially over time. All in all, epidemiological estimates of injury in women's professional football are likely influenced by a variety of contextual and methodological factors. Therefore, these factors must be considered when assessing data, for example, from different countries, regions, levels of competition, and collection methods.

The relatively small number of injuries reported in previous studies, largely due to small sample sizes, limits the external validity of the estimates of injury incidence and burden and hinders meaningful exploration of the data to identify common injury patterns and diagnoses. Moreover, given the significant negative consequences of certain injuries in women's football, such as the high burden of anterior cruciate ligament injuries (ACL) (38.0 days lost/1000 h, [8]), more up‐to‐date investigations across different populations are required. Accurate and context‐specific estimates of injury occurrence and severity are essential to enable practitioners to appropriately monitor and improve the health and performance of elite female football players within a specific context (i.e., population), as well as optimizing the development of epidemiological research.

Therefore, the aim of this study was to provide a surveillance of injury incidence, severity, burden, cause, onset, and to explore patterns of injury (type, location, specific diagnoses) across five seasons (2018–2023) during match‐play and training in the top two divisions of English Women's domestic football.

## 2 Materials and Methods

### 2.1 Study Design

The data presented here contains results from the *FA Injury and Illness Surveillance Programme*, which collects, analyzes, and interprets injury and illness data across English football. The present study is a 5‐year prospective cohort study, including data collected from 2018 to 2023 in teams playing in the top two tiers of English Women's football (FA Women's Super League and Women's Championship). Due to the impact of the COVID‐19 pandemic, the 2019–2020 season was curtailed; therefore, data from match‐play for this season was only collected until 13 March 2020. The retrospective analyses of data were approved by the Nottingham Trent University Non‐Invasive Ethical Review Committee (application number: 116V2) and informed consent was obtained from all clubs prior to data collection.

### 2.2 Participants

Players included in the present study were contracted to the clubs competing in the two English women's professional leagues, with teams providing injury and exposure data from consenting players. Data included was related to injuries and exposure that occurred in club‐directed training and match‐play. Injuries and exposure that took place during non‐club related activity were not included in the present study. There were 93 individual team seasons, 48 Women's Super League team seasons, and 45 Women's Championship team seasons, with the number of teams analyzed per season shown in Table 1.

**TABLE 1:** Number of participating teams and number of teams reporting complete match and training exposure data.

	Season					Total
	18/19	19/20	20/21	21/22	22/23	
Total teams (*n*)	9	19	20	23	22	93
Teams providing match exposure (*n*)	9	19	20	23	22	93
Match exposure (h)	3993	6575	8742	11 641	12 069	43 020
Match injuries (*n*)	110	165	212	244	250	981
Teams providing training exposure (*n*)	0	4	10	15	18	47
Training exposure (h)	N/A	10 415	33 912	63 448	104 477	212 252
Training injuries (*n*)	109	204	292	308	273	1186

Abbreviations: h, hours; *n*, number of teams.

### 2.3 Injury and Exposure Data Collection

In accordance with consensus guidelines [18, 19], a time‐loss injury was considered to have occurred when a training or match‐play incident prevented a player being available for 1 or more days [18, 19]. Raw injury data and corresponding days absent were reported by club medical staff using their preferred medical record system. Data were retrospectively categorized according to the type of event (match/training/other club related), severity (minor = 1–7 days absent, moderate = 8–28 days, severe = 29–89 days, major = ≥ 90 days), cause (contact, non‐contact, cumulative), onset (acute, gradual), type, location and diagnosis, according to the Olympic Committee [20] and football [18, 19] consensus statements, and previous research for the *FA Injury and Illness Surveillance Programme* [21].

Match exposure was recorded as the total minutes played per player during all league and cup fixtures. Where match exposure data was not provided, corresponding match injury data could not be included in the incidence and burden analysis. The weekly training exposure for each team was calculated using the formula: Training exposure per week = duration of training sessions per week × number of players involved. Exposure time was provided by the club's preferred medical record systems or sent directly to research staff via a club‐based practitioner. To facilitate the accurate recording of data, written guidance in the form of bespoke documents created by the FA and others [22], and individual guidance was given to each team's medical support staff, who were able to send queries to the research team via email.

### 2.4 Data Analysis

Injury incidence is reported as the number of injuries per 1000 h of exposure, burden is the total number of days absent per 1000 h of exposure, and median severity has been presented as median (IQR).

Incidence and burden rate estimates were reported with accompanying 95% confidence intervals (95% CI), determined by a clustered, bootstrapped negative binomial approach. First, teams are resampled with replacement, followed by resampling observations within each selected team to account for hierarchical clustering. For each bootstrapped dataset, exposure values are obtained directly from the observed data, while injury counts are simulated 10 000 times using a negative binomial distribution to account for overdispersion (overdispersion parameter, *θ* = 20). To determine differences between overall match and training incidence and burden, an injury rate ratio (IRR) with associated 95% confidence intervals was calculated, employing a similar clustered, negative binomial bootstrap procedure. Effect estimates with associated 95% confidence intervals excluding 1.00 indicated statistical significance.

Time‐trend analysis was performed employing a similar clustered, bootstrapped negative binomial approach. The analysis was performed to determine changes in match and training incidence and burden across the study period for all injuries and hamstring injuries. A log‐linear negative binomial model was used within each bootstrap iteration to estimate yearly trends, where the slope coefficient (*β*) represents the log of the multiplicative change in injury rate per year. The yearly percentage change was calculated as (exp(*β*)–1)*100, with statistical significance set at *p* < 0.05. Given the low instances of knee anterior cruciate ligament (ACL) injuries, changes in match injury incidence and burden over time were analyzed using IRR (95% CI) whereby the 2018–2019 to 2021–2022 seasons were aggregated and compared to the 2022–2023 season. Due to a low number of instances, of which were lower than in matches, changes in ACL training incidence and burden over time were not statistically analyzed.

The incidence and burden estimates for type and location of injury with accompanying 95% confidence interval were derived from aggregated datasets using a parametric negative binomial model with bootstrapped confidence intervals simulated from the model. Injury counts were simulated 10 000 times from a negative binomial distribution to account for overdispersion (overdispersion parameter, *θ* = 20). All analyses determining incidence and burden estimates, 95% confidence intervals, time‐trend analysis, and injury rate ratios (IRR) were performed using the open access R software package (www.r‐project.org).

The chi‐square (*χ* ^2^) test of independence was performed using Microsoft Excel (Microsoft Windows 10, 2018) to determine differences in the distribution of match and training injuries for injury severity, cause, onset, type, location, and diagnoses. Statistical significance was set at *p* < 0.05.

## 3 Results

Over five seasons in English women's domestic football, 2167 injuries were reported; 981 (45%) occurred in matches, and 1186 (55%) occurred in training. There was a total of 76 687 days absent recorded, 37 269 days absent due to match injuries, and 39 418 days absent due to training injuries. Total exposure over the study period seasons was 255 272 h. Match exposure over the five seasons was 43 020 h and training exposure over four seasons was 212 252 h (Table 1).

Where match and training exposure data were not provided, corresponding injury data could not be included in the incidence and burden analysis. There were 98 match injuries, 10% of all injuries, excluded from the incidence and burden calculations (2018–2019 = 1; 2019–2020 = 19; 2020–2021 = 19; 2021–2022 = 28; 2022–2023 = 31). From 2019–2020 to 2022–2023, 4, 10, 15, and 18 teams offered training exposure. There were 470 training injuries, 44% of all injuries, excluded from the incidence and burden calculations (2019–2020 = 135; 2020–2021 = 153; 2021–2022 = 131; 2022–2023 = 51), with 100% of injuries removed from the 2018–2019 season given no teams provided training exposure.

### 3.1 Match and Training Injury Incidence and Burden

The incidence of injury in match‐play (20.5 injuries/1000 h [95% CI: 16.9–24.1]) was greater than training (2.9 injuries/1000 h [95% CI: 2.0–4.0]) (IRR: 7.3, 95% CI: 5.1–10.1). Time‐trend analyses showed a 28% decrease in training injury incidence across the study period (*β* = −0.33, *p* = 0.041) but no change in match injury incidence (−8%, *β* = −0.08, *p* = 0.218) (Table 2).

**TABLE 2:** Match and training injury incidence and burden from 2018–2019 to 2022–2023.

	Match				Training			
	No. of injuries/team	Incidence [injuries/1000 h (95% CI)]	Median severity [days (IQR)]	Burden [days absent/1000 h (95% CI)]	No. of injuries/team	Incidence [injuries/1000 h (95% CI)]	Median severity [days (IQR)]	Burden [days absent/1000 h (95% CI)]
2018–2019	12	27.3 (17.1–37.6)	17 (6–35)	789.9 (418.8–1285.4)	N/A	N/A	15 (6–33)	N/A
2019–2020	8	22.2 (14.7–30.3)	14 (6–34)	840.9 (538.9–1160.9)	17	6.8 (2.9–12.7)	12 (5–34)	193.7 (44.5–333.7)
2020–2021	10	22.0 (14.6–30.1)	12 (5–35)	655.4 (470.3–863.4)	14	4.2 (2.1–7.2)	17 (6–38)	123.1 (71.7–185.5)
2021–2022	9	18.5 (13.4–24.4)	13 (6–34)	697.9 (477.3–933.1)	12	2.9 (1.7–4.5)	13 (5–33)	98.4 (55.1–169.0)
2022–2023	10	18.2 (12.3–24.6)	17 (6–41)	893.4 (572.3–1325.1)	15	2.2 (1.3–3.3)	13 (6–42)	79.7 (45.3–130.3)
Average (95% CI)	10 (8–12)	20.5^b^ (16.9–24.1)	14 (6–36)	775.1^b^ (630.0–934.9)	15 (11–18)	2.9 (2.0–4.0)	13 (6–37)	96.9 (66.1–138.5)
Seasonal change (%)	—	−8	—	2	—	−28^a^	—	−19
*p*	—	0.218	—	0.393	—	0.041	—	0.179

*Note:* The number of injuries/team, incidence, and burden presented have been adapted for missing corresponding exposure, with injuries excluded from the calculations where required.

Abbreviation: IQR, interquartile range.

^a^ Significant seasonal change in injury incidence from 2018–2019 to 2022–2023 (*p* < 0.05).

^b^ Significant difference between match and training (*p* < 0.05); N/A = no training exposure provided for this season, therefore training injury incidence and burden could not be calculated.

The burden of injury in match‐play (775.1 days absent/1000 h [95% CI: 630.0–934.9]) was greater than training (96.9 days absent/1000 h [95% CI: 66.1–138.5]) (IRR: 8.3, 95% CI: 5.4–12.0). However, there were no significant changes in injury burden in match‐play (2%, *β* = 0.02, *p* = 0.393) or training (−19%, *β* = −0.21, *p* = 0.179) across the study period (Table 2).

### 3.2 Severity, Cause, and Onset of Injury

Overall, most injuries reported were of a moderate severity (8–28 days, 37%), with the highest proportion of injuries occurring via contact mechanisms (56%) and acute onset (79%). There was no difference in the severity of injury between match‐play and training (*χ* ^2^[3,*N* = 2152] = 1.97, *p* = 0.579). Contact injuries were more common in match‐play compared to training, while non‐contact injuries were more common in training (*χ* ^2^[1,*N* = 1734] = 84.4, *p* < 0.001). Acute injuries were more common in match‐play compared to training, while gradual injuries were more common in training (*χ* ^2^[1,*N* = 1796] = 21.8, *p* < 0.001) (Table 3).

**TABLE 3:** Total number of injuries according to severity, cause, and onset.

	Total no. of injuries (*n*, %)	Match total no. of injuries (*n*, %)	Training no. of injuries (*n*, %)
**Severity**	**(*n* = 2152)**	**(*n* = 974)**	**(*n* = 1178)**
Minor	703 (33%)	311 (32%)	392 (33%)
Moderate	791 (37%)	366 (38%)	425 (36%)
Severe	469 (22%)	205 (21%)	264 (22%)
Major	189 (9%)	92 (9%)	97 (8%)
**Cause**	**(*n* = 1734)**	**(*n* = 808)**	**(*n* = 926)**
Contact	975 (56%)	549 (68%)^a^	426 (46%)
Non‐contact	759 (44%)	259 (32%)	500 (54%)^b^
Cumulative	0 (0%)	0 (0%)	0 (0%)
**Onset**	**(*n* = 1796)**	**(*n* = 834)**	**(*n* = 962)**
Acute	1421 (79%)	700 (84%)^a^	721 (75%)
Gradual	375 (21%)	134 (16%)	241 (25%)^b^

Abbreviation: *N*, number of injuries.

^a^ Greater proportion in match‐play compared to training.

^b^ Greater proportion in training compared to match‐play (all *p* < 0.05).

### 3.3 Type of Injury

Muscle and ligament injuries were the most common types of injury in both match‐play and training (Table 4). There were notable differences in the type of injury between match‐play and training; whereby the distribution of muscle and tendon injuries that occurred in training were 35% (*χ* ^2^[1,*N* = 2167] = 19.02, *p* < 0.001) and 43% (*χ* ^2^[1,*N* = 2167] = 3.93, *p* = 0.048) greater compared to match‐play, respectively (Table 4).

**TABLE 4:** Descriptive data for type of injury that occurred overall, in match‐play and training.

	Injury														
Type	Frequency (*n*, % of total)			Incidence [injuries/1000 h (95% CI)]			Median severity [days (IQR)]			Total severity [days, (%)]			Burden [days absent/1000 h (95% CI)]		
	All	Match	Training	All	Match	Training	All	Match	Training	All	Match	Training	All	Match	Training
Muscle strain/tear/rupture/cramps	620 (28.6%)	235 (24.0%)	385 (32.5%)^b^	1.60 (0.52–3.24)	4.88 (1.60–10.02)	0.93 (0.30–1.95)	12 (6–25)	13 (6–27)	11 (5–23)	13 076 (17.1%)	5422 (14.5%)	7654 (19.4%)^b^	36.27 (11.78–73.38)	118.48 (38.54–238.01)	19.60 (6.38–40.57)
Ligament sprain/tear/rupture	395 (18.2%)	190 (19.4%)	205 (17.3%)	1.09 (0.35–2.27)	3.79 (1.19–7.83)	0.54 (0.17–1.12)	20 (6–51)	29 (9–65)	15 (6–41)	23 029 (30.0%)	13 692 (36.7%)^a^	9337 (23.7%)	65.68 (21.43–134.72)	266.83 (88.61–552.26)	24.91 (8.05–51.47)
Tendon injury/rupture/tendinosis	118 (5.4%)	43 (4.4%)	75 (6.3%)^b^	0.33 (0.10–0.69)	0.91 (0.26–1.93)	0.22 (0.06–0.46)	18 (6–44)	12 (6–31)	21 (7–55)	4488 (5.9%)	1369 (3.7%)	3119 (7.9%)^b^	12.42 (3.99–25.79)	30.17 (9.72–63.16)	8.82 (2.85–18.22)
Haematoma/contusion/bruise	151 (7.0%)	100 (10.2%)^a^	51 (4.3%)	0.48 (0.15–1.00)	2.21 (0.67–4.58)	0.13 (0.03–0.29)	5 (3–8)	5 (3–8)	6 (3–13)	1415 (1.8%)	778 (2.1%)^a^	637 (1.6%)	4.20 (1.37–8.68)	17.55 (5.51–36.31)	1.49 (0.49–3.06)
Concussion	116 (5.4%)	66 (6.7%)^a^	50 (4.2%)	0.33 (0.10–0.70)	1.44 (0.42–3.00)	0.10 (0.02–0.23)	11 (6–19)	12 (7–20)	10 (6–18)	1744 (2.3%)	993 (2.7%)^a^	751 (1.9%)	4.98 (1.62–10.24)	22.52 (7.18–45.37)	1.42 (0.46–2.87)
Bursitis/impingement/synovitis	38 (1.8%)	13 (1.3%)	25 (2.1%)	0.09 (0.02–0.20)	0.26 (0.05–0.58)	0.06 (0.01–0.14)	18 (8–48)	13 (7–32)	19.5 (9–52)	1365 (1.8%)	410 (1.1%)	955 (2.4%)^b^	2.58 (0.81–5.34)	6.65 (2.14–13.51)	1.76 (0.56–3.54)
Cartilage/disc/meniscus	76 (3.5%)	29 (3.0%)	47 (4.0%)	0.22 (0.06–0.45)	0.63 (0.16–1.32)	0.13 (0.03–0.28)	31 (11–73)	25.5 (9–63)	35 (12–76)	4195 (5.5%)	1707 (4.6%)	2488 (6.3%)^b^	11.13 (3.56–22.66)	34.59 (11.13–70.92)	6.38 (2.03–13.12)
Bone fracture	69 (3.2%)	33 (3.4%)	36 (3.0%)	0.22 (0.07–0.45)	0.74 (0.21–1.60)	0.11 (0.03–0.23)	57 (30–90)	60 (27–97)	52 (33–89)	4491 (5.9%)	2213 (5.9%)	2278 (5.8%)	14.04 (4.55–28.7)	47.56 (15.55–98.44)	7.25 (2.28–14.53)
Pain (undiagnosed)	55 (2.5%)	18 (1.8%)	37 (3.1%)	0.17 (0.05–0.36)	0.42 (0.09–0.91)	0.12 (0.03–0.25)	8 (5–20)	11 (4–21)	6 (5–18)	859 (1.1%)	386 (1.0%)	473 (1.2%)^b^	2.73 (0.89–5.57)	8.97 (2.84–18.41)	1.47 (0.46–2.99)
Other	524 (24.2%)	250 (25.5%)	274 (23.1%)	1.30 (0.42–2.64)	5.18 (1.63–10.60)	0.51 (0.16–1.04)	15.5 (6–46)	13 (6–44)	17 (6–50)	21 921 (28.6%)	10 228 (27.4%)	11 693 (29.7%)^b^	56.36 (18.52–115.14)	219.57 (69.15–452.16)	23.27 (7.38–48.07)
Unspecified	5 (0.2%)	4 (0.4%)	1 (0.1%)	0.02 (0.00–0.04)	0.07 (0.00–0.19)	0.005 (0.00–0.02)	23 (8–33)	15.5	—	104 (0.1%)	71 (0.2%)^a^	33 (0.1%)	0.38 (0.11–0.77)	1.46 (0.44–3.02)	—

*Note:* Frequency of injuries, median severity [days lost, IQR] and total severity [days lost, %] represent all injuries reported. Median severity and burden are only reported for seasons presenting more than one injury. IQR for severity is only reported for seasons presenting five or more injuries. The incidence and burden presented have been adapted for missing corresponding exposure, with injuries excluded from the calculations where required.

^a^ Greater proportion of match compared to training injuries.

^b^ Greater proportion of training compared to match injuries; determined via chi‐square analysis highlighting differences between the proportion of match and training injuries for frequency and severity, where *p* < 0.05.

Ligament and muscle injuries were the most burdensome in both match‐play and training (Table 4). The proportion of days absent for muscle and tendon injuries that occurred in training was 34% (*χ* ^2^[1,*N* = 76 687] = 321.13, *p* < 0.001) and 114% (*χ* ^2^[1,*N* = 76 687] = 624.85, *p* < 0.001) greater compared to match‐play, respectively, whereas the proportion of days absent for ligament injuries occurring in matches was 55% greater than training (*χ* ^2^[1,*N* = 76 687] = 1552.93, *p* < 0.001) (Table 4).

### 3.4 Location of Injury

The thigh and knee were the most common injury locations in both matches and training (Table 5). There were notable differences in the proportion of injuries for location between matches and training. Achilles tendon (186%, *χ* ^2^[1, *N* = 2167] = 6.53, *p* = 0.011) and thigh injuries that occurred in training (32%, *χ* ^2^[1, *N* = 2167] = 13.45, *p* < 0.001) were greater compared to matches, whereas head/face injuries that occurred in matches were greater compared to training (72%, *χ* ^2^[1, *N* = 2167] = 14.63, *p* < 0.001) (Table 5).

**TABLE 5:** Frequency and incidence of location and diagnoses of injury that occurred overall, in matches and training.

Location	Injury					
*Diagnosis*	Frequency (% of total)			Incidence [injuries/1000 h (95% CI)]		
	All	Match	Training	All	Match	Training
Abdomen	10 (0.5%)	4 (0.4%)	6 (0.5%)	0.02 (0.00–0.05)	0.07 (0.00–0.19)	0.01 (0.00–0.03)
Achilles tendon	31 (1.4%)	7 (0.7%)	24 (2.0%)^b^	0.07 (0.02–0.16)	0.14 (0.02–0.33)	0.06 (0.01–0.13)
Ankle	390 (18.0%)	187 (19.1%)	203 (17.1%)	1.02 (0.33–2.12)	3.72 (1.16–7.72)	0.48 (0.15–0.99)
*Lateral ankle ligament*	129 (6.0%)	57 (5.8%)	72 (6.1%)	0.33 (0.10–0.68)	1.07 (0.30–2.19)	0.17 (0.05–0.37)
Arm	21 (1.0%)	7 (0.7%)	14 (1.2%)	0.06 (0.02–0.14)	0.14 (0.02–0.35)	0.05 (0.01–0.11)
Foot/toe	129 (6.0%)	69 (7.0%)	60 (5.1%)	0.38 (0.12–0.80)	1.49 (0.46–3.11)	0.16 (0.05–0.33)
Head/face	184 (8.5%)	108 (11.0%)^a^	76 (6.4%)	0.52 (0.16–1.06)	2.30 (0.70–4.77)	0.16 (0.04–0.35)
*Concussion*	116 (5.4%)	66 (6.7%)^a^	50 (4.2%)	0.33 (0.10–0.70)	1.46 (0.44–3.00)	0.10 (0.03–0.23)
*Head/face/neck injury*	83 (3.8%)	50 (5.1%)^a^	33 (2.8%)	0.23 (0.07–0.47)	1.00 (0.28–2.14)	0.07 (0.01–0.16)
Hip/groin	90 (4.2%)	40 (4.1%)	50 (4.2%)	0.23 (0.07–0.49)	0.86 (0.23–1.84)	0.10 (0.02–0.23)
*Hip/groin muscle/tendon*	25 (1.2%)	10 (1.0%)	15 (1.3%)	0.05 (0.01–0.10)	0.21 (0.05–0.49)	0.01 (0.00–0.04)
Knee	403 (18.6%)	191 (19.5%)	212 (17.9%)	1.11 (0.35–2.28)	4.09 (1.28–8.41)	0.51 (0.16–1.06)
*Anterior cruciate (ACL)*	35 (1.6%)	22 (2.2%)^a^	13 (1.1%)	0.11 (0.03–0.24)	0.49 (0.12–1.05)	0.03 (0.00–0.08)
*Medial collateral (MCL)*	56 (2.6%)	26 (2.7%)	30 (2.5%)	0.16 (0.05–0.35)	0.56 (0.14–1.21)	0.08 (0.02–0.19)
*Meniscal/cartilage*	52 (2.4%)	23 (2.3%)	29 (2.4%)	0.15 (0.04–0.31)	0.51 (0.12–1.12)	0.08 (0.02–16)
Low back	60 (2.8%)	20 (2.0%)	40 (3.4%)	0.14 (0.04–0.31)	0.40 (0.09–0.88)	0.09 (0.02–0.20)
Lower Leg	169 (7.8%)	81 (8.3%)	88 (7.4%)	0.47 (0.14–0.95)	1.84 (0.56–3.84)	0.19 (0.05–0.40)
*Triceps surae*	93 (4.3%)	41 (4.2%)	52 (4.4%)	0.26 (0.08–0.54)	0.93 (0.26–1.95)	0.13 (0.03–0.28)
Neck/cervical spine	15 (0.7%)	8 (0.8%)	7 (0.6%)	0.04 (0.01–0.09)	0.16 (0.02–0.37)	0.01 (0.00–0.04)
Pelvis/sacrum	27 (1.2%)	9 (0.9%)	18 (1.5%)	0.06 (0.02–0.14)	0.21 (0.05–0.49)	0.03 (0.00–0.08)
Shoulder/clavicle	37 (1.7%)	24 (2.4%)^a^	13 (1.1%)	0.12 (0.03–0.26)	0.53 (0.14–1.14)	0.04 (0.00–0.09)
Sternum/ribs/upper back	9 (0.4%)	3 (0.3%)	6 (0.5%)	0.02 (0.00–0.04)	0.05 (0.00–0.14)	0.01 (0.00–0.03)
Thigh	550 (25.4%)	212 (21.6%)	338 (28.5%)^b^	1.44 (0.47–2.99)	4.30 (1.35–8.83)	0.86 (0.27–1.79)
*Hamstring muscle/tendon*	253 (11.7%)	108 (11.0%)	145 (12.2%)	0.72 (0.23–1.49)	2.37 (0.74–4.90)	0.38 (0.12–0.79)
*Quadriceps muscle/tendon*	155 (7.2%)	46 (4.7%)	109 (9.2%)^b^	0.37 (0.12–0.77)	0.79 (0.21–1.67)	0.28 (0.08–0.58)
*Adductor muscle/tendon*	66 (3.0%)	20 (2.0%)	46 (3.9%)^b^	0.14 (0.04–0.30)	0.40 (0.09–0.86)	0.09 (0.02–0.20)
Wrist/hand/finger/thumb	34 (1.6%)	6 (0.6%)	28 (2.4%)^b^	0.09 (0.02–0.19)	0.14 (0.02–0.35)	0.08 (0.02–0.17)
Unspecified	8 (0.4%)	5 (0.5%)	3 (0.3%)	0.03 (0.00–0.07)	0.09 (0.00–0.23)	0.01 (0.00–0.04)

*Note:* Frequency of injuries represent all injuries reported. Specific injury locations and diagnosis have only been presented if five or more injuries occurred overall. The incidences presented have been adapted for missing corresponding exposure, with injuries excluded from the calculations where required. All lower limb muscle/tendon injuries do not include haematomas/contusions/bruises.

^a^ Greater proportion of match compared to training injuries.

^b^ Greater proportion of training compared to match injuries; determined via chi‐square analysis highlighting differences between the proportion of match and training injuries for frequency and severity, where *p* < 0.05.

Knee and ankle injuries were the most burdensome in matches, while knee and thigh injuries were the most burdensome in training (Table 6). The proportion of days absent for Achilles tendon (117%, *χ* ^2^[1, *N* = 76 687] = 197.94, *p* < 0.001), ankle (5%, *χ* ^2^[1, *N* = 76 687] = 6.59, *p* = 0.010) and thigh injuries that occurred in training (48%, *χ* ^2^[1, *N* = 76 687] = 478.66, *p* < 0.001) were greater compared to matches, whereas the proportion of days absent for knee injuries that occurred in matches was greater compared to training (49%, *χ* ^2^[1, *N* = 76 687] = 2097.13, *p* < 0.001) (Table 6).

**TABLE 6:** Total severity, median severity and injury burden of location and diagnoses of injury that occurred overall, in matches and training.

Location	Injury								
*Diagnosis*	Total severity [days lost (% of total)]			Median severity [days lost (IQR)]			Burden [days absent/1000 h (95% CI)]		
	All	Match	Training	All	Match	Training	All	Match	Training
Abdomen	219 (0.3%)	147 (0.4%)^a^	72 (0.2%)	7 (4–15)	10	7 (5–12)	0.56 (0.18–1.15)	3.05 (0.95–6.32)	0.06 (0.01–0.14)
Achilles tendon	1469 (1.9%)	447 (1.2%)	1022 (2.6%)^b^	19 (7–59)	42 (21–52)	15 (6–70)	4.24 (1.37–8.71)	9.34 (2.91–19.43)	3.20 (1.03–6.60)
Ankle	12 067 (15.7%)	5735 (15.4%)	6332 (16.1%)^b^	17 (6–41)	18.5 (6–41)	16 (6–41)	31.14 (10.01–64.46)	113.06 (36.89–232.40)	14.53 (4.67–29.34)
*Lateral ankle ligament*	2857 (3.7%)	1436 (3.9%)	1421 (3.6%)	13 (5–29)	23 (5–34)	9 (5–22)	6.94 (2.24–14.3)	24.15 (7.97–49.91)	3.45 (1.11–7.12)
Arm	754 (1.0%)	112 (0.3%)	642 (1.6%)^b^	13 (4–52)	13 (5–18)	13 (5–71)	2.44 (0.76–4.89)	2.30 (0.72–4.74)	2.46 (0.80–5.09)
Foot/toe	5407 (7.1%)	2411 (6.5%)	2996 (7.6%)^b^	20 (5–60)	8.5 (4–51)	32.5 (9–71)	15.36 (4.88–31.66)	48.21 (15.83–99.51)	8.70 (2.84–17.94)
Head/face	2494 (3.3%)	1492 (4.0%)^a^	1002 (2.5%)	10 (6–17)	11 (6–18)	9 (6–15)	7.02 (2.27–14.31)	32.66 (10.67–66.46)	1.82 (0.58–3.76)
*Concussion*	1744 (2.3%)	993 (2.7%)^a^	751 (1.9%)	11 (6–19)	12 (7–20)	10 (6–18)	5.00 (1.63–10.23)	22.66 (7.21–46.21)	1.42 (0.45–2.95)
*Head/face/neck injury*	964 (1.3%)	551 (1.5%)^a^	413 (1.0%)	6 (3–14)	8 (2–15)	6 (3–13)	2.70 (0.89–5.55)	11.02 (3.56–22.66)	1.01 (0.32–2.10)
Hip/groin	2323 (3.0%)	1015 (2.7%)	1308 (3.3%)^b^	11 (6–21)	11 (6–19)	10 (5–25)	5.96 (1.91–12.07)	18.78 (6.16–38.01)	3.36 (1.06–6.79)
*Hip/groin muscle/tendon*	464 (0.6%)	215 (0.6%)	249 (0.6%)	10 (6–17)	12 (10–18)	8 (5–15)	1.17 (0.39–2.39)	4.90 (1.56–10.20)	0.41 (0.13–0.87)
Knee	31 612 (41.2%)	18 483 (49.6%)^a^	13 129 (33.3%)	28 (9–83)	37 (9–123)	24 (9–62)	88.69 (29.49–181.40)	384.57 (123.90–776.06)	28.73 (9.36–59.87)
*Anterior cruciate (ACL)*	10 216 (13.3%)	6222 (16.7%)^a^	3994 (10.1%)	338 (283–365)	338 (292–363)	338 (283–390)	32.00 (10.52–66.56)	136.31 (45.42–277.32)	10.86 (3.57–22.20)
*Medial collateral (MCL)*	1918 (2.5%)	1105 (3.0%)^a^	813 (2.1%)	30 (10–52)	42.5 (20–61)	18 (7–36)	6.06 (1.94–12.54)	24.17 (7.86–50.00)	2.39 (0.80–4.88)
*Meniscal/cartilage*	2837 (3.7%)	1309 (3.5%)	1528 (3.9%)^b^	32 (13–72)	20.5 (7–43)	38 (15–76)	8.31 (2.59–16.82)	29.80 (9.46–61.13)	3.95 (1.29–8.07)
Low back	1223 (1.6%)	393 (1.1%)	830 (2.1%)^b^	9 (5–31)	10 (5–25)	7.5 (5–31)	2.51 (0.81–5.04)	8.51 (2.77–17.43)	1.30 (0.42–2.67)
Lower leg	4157 (5.4%)	1574 (4.2%)	2853 (6.6%)^b^	15 (7–32)	12 (6–23)	19 (8–39)	11.57 (3.67–23.69)	35.87 (11.92–73.90)	6.65 (2.14–13.51)
*Triceps surae*	2149 (2.8%)	833 (2.2%)	1316 (3.3%)^b^	18 (7–32)	17 (6–24)	20 (8–37)	6.47 (2.09–13.36)	18.90 (6.25–38.57)	3.95 (1.28–8.09)
Neck/cervical spine	214 (0.3%)	52 (0.1%)	162 (0.4%)^b^	4 (2–8)	3.5 (2–7)	4 (3–13)	0.68 (0.22–1.39)	1.02 (0.30–2.12)	0.61 (0.19–1.25)
Pelvis/sacrum	615 (0.8%)	88 (0.2%)	527 (1.3%)^b^	7 (5–19)	7 (6–12)	7.5 (3–24)	0.75 (0.23–1.54)	2.05 (0.63–4.21)	0.49 (0.16–1.00)
Shoulder/clavicle	1494 (1.9%)	752 (2.0%)	742 (1.9%)	25 (12–55)	24.5 (12–42)	25 (10–92)	4.54 (1.49–9.17)	16.57 (5.39–34.84)	2.10 (0.66–4.37)
Sternum/ribs/upper back	174 (0.2%)	46 (0.1%)	128 (0.3%)^b^	20 (7–23)	8	20 (10–22)	0.44 (0.14–0.92)	0.95 (0.28–2.02)	0.34 (0.10–0.70)
Thigh	11 194 (14.6%)	4371 (11.7%)	6823 (17.3%)^b^	11 (5–23)	11.5 (6–27)	11 (5–21)	31.30 (10.17–64.52)	94.09 (30.24–188.62)	18.57 (6.04–37.21)
*Hamstring muscle/tendon*	6370 (8.3%)	2828 (7.6%)	3542 (9.0%)^b^	14 (7–31)	16.5 (8–33)	13 (6–30)	18.56 (6.01–38.10)	63.97 (20.90–130.82)	9.35 (3.03–19.13)
*Quadriceps muscle/tendon*	2781 (3.6%)	882 (2.4%)	1899 (4.8%)^b^	10 (5–20)	10.5 (5–19)	10 (5–20)	7.29 (2.37–14.96)	16.71 (5.35–33.82)	5.39 (1.72–11.19)
*Adductor muscle/tendon*	970 (1.3%)	269 (0.7%)	701 (1.8%)^b^	9 (4–19)	7.5 (4–22)	10 (4–17)	1.97 (0.63–4.02)	5.30 (1.63–10.76)	1.30 (0.41–2.67)
Wrist/hand/finger/thumb	800 (1.0%)	78 (0.2%)	722 (1.8%)^b^	17 (6–33)	8 (7–20)	18 (6–40)	1.77 (0.54–3.60)	1.81 (0.53–3.77)	1.76 (0.56–3.70)
Unspecified	471 (0.6%)	73 (0.2%)	398 (1.0%)^b^	28 (7–66)	8 (4–23)	156	1.81 (0.56–3.70)	1.51 (0.46–3.16)	1.88 (0.59–3.84)

*Note:* Median severity [days lost, IQR] and total severity [days lost, %] represent all injuries reported. Specific injury locations and diagnoses have only been presented if five or more injuries occurred overall. Median severity and burden are only reported for seasons presenting more than one injury. IQR for severity is only reported for seasons presenting five or more injuries. The burdens presented have been adapted for missing corresponding exposure, with injuries excluded from the calculations where required. All lower limb muscle/tendon injuries do not include haematomas/contusions/bruises.

^a^ Greater proportion of match compared to training injuries.

^b^ Greater proportion of training compared to match injuries; determined via chi‐square analysis highlighting differences between the proportion of match and training injuries for frequency and severity, where *p* < 0.05.

### 3.5 Diagnoses of Injury

ACL match injury incidence from 2018–2022 was 0.35 injuries/1000 h (95% CI 0.06–0.77), while in 2022–2023 it was 0.80 injuries/1000 h (95% CI 0.07–1.98). No significant change was observed between the two periods (IRR: 0.71, 95% CI: 0.07–2.76). Ninety‐three team seasons were assessed, 76 (82%) of the team seasons recorded zero ACL injuries, 15 (16%) recorded one occurrence, and two (2%) recorded more than one occurrence. Hamstring muscle/tendon injuries were the most common injury diagnosis in matches (2.37 injuries/1000 h [95% CI: 0.74–4.90]) and training (0.38 injuries/1000 h [95% CI: 0.12–0.79]) (Table 5). There were no significant seasonal changes in hamstring injury incidence in match‐play (−5%, *β* = −0.05, *p* = 0.480) or training (−10%, *β* = −0.10, *p* = 0.412).

There were notable differences in the proportion of injuries for diagnoses between matches and training. Quadriceps (96%, *χ* ^2^[1, *N* = 2167] = 16.38, *p* < 0.001) and adductor injuries that occurred in training (95%, *χ* ^2^[1, *N* = 2167] = 6.15, *p* = 0.013) were greater compared to matches, whereas concussion (60%, *χ* ^2^[1, *N* = 2167] = 6.69, *p* = 0.010) and ACL injuries that occurred in matches (100%, *χ* ^2^[1, *N* = 2167] = 4.44, *p* = 0.035) were greater compared to training (Table 5).

Knee ACL and hamstring muscle/tendon injuries were the most burdensome injury diagnoses in both matches and training (Table 6). ACL match injury burden from 2018–2022 was 91.88 days lost/1000 h (95% CI: 24.16–179.50), while in 2022–2023 it was 249.88 days lost/1000 h (95% CI: 39.75–566.63). No significant change was observed between the two periods (IRR: 0.54, 95% CI: 0.08–1.89). There were no significant seasonal changes in hamstring injury burden in match‐play (0%, *β* = 0, *p* = 0.278) or training (−2%, *β* = −0.01, *p* = 0.438).

The proportion of days absent for hamstring (18%, *χ* ^2^[1, *N* = 76 687] = 49.13, *p* < 0.001), quadriceps (100%, *χ* ^2^[1, *N* = 76 687] = 329.29, *p* < 0.001) and adductor injuries that occurred in training (157%, *χ* ^2^[1, *N* = 76 687] = 171.24, *p* < 0.001) were greater compared to matches. The proportion of days absent for concussion (42%, *χ* ^2^[1, *N* = 76 687] = 49.68, *p* < 0.001) and ACL injuries that occurred in matches (65%, *χ* ^2^[1, *N* = 76 687] = 714.46, *p* < 0.001) was greater compared to training (Table 6).

## 4 Discussion

This study presents data on 2167 injuries across 5 seasons of English women's domestic football, making it the largest injury surveillance study in English professional women's football, and provides useful benchmarks for injury incidence, burden, and patterns. Injury incidence and burden were higher in match‐play than in training (incidence: 20.5 vs. 2.9 injuries/1000 h; burden: 775.1 vs. 96.9 days absent/1000 h). Training injury incidence decreased by 28% across the study period, but no change was observed for match injury incidence or injury burden. ACL injury accounted for a small proportion of total injuries (1.6%) but had the highest percentage of days missing due to injury compared to other diagnoses (13.3%), by virtue of its high burden (32.00 days absent/1000 h). Hamstring muscle/tendon injuries were the most common injury diagnosis, accounting for 0.72/1000 h, and caused the second highest injury burden (18.56 days absent/1000 h). There were no significant seasonal changes in ACL or hamstring injury incidence or burden.

### 4.1 Injury Incidence and Burden

There was a 28% reduction in training injury incidence across the study period, and a non‐significant 8% decrease in match injury incidence, whereas no changes were observed for injury burden. In contrast, the most recent season for which data are available in the UEFA WECIS (2021–2022) was reported to have a greater training injury incidence than the 2018–2019 season, and a greater match injury incidence than the 2019–2020 season, albeit representing a COVID‐19 affected season [8]. Training injury incidence in the present study (2.9/1000 h) was lower than the UEFA WECIS (4.8/1000 h) [8], but similar to pooled estimates from women's domestic competitions from 1988 to 2015 (3.2/1000 h) [6]. Meanwhile, match injury incidence in the current study (20.5/1000 h) is slightly higher than reported in the UEFA WECIS (18.4/1000 h) and pooled estimates in women's football (19.5/1000 h) [6]. Injury burden for match‐play (775.1 days absent/1000 h) was higher than previously reported in the UEFA WECIS (537.3/1000 h) but training (96.9 days absent/1000 h) was similar (UEFA WECIS: 110.1) [8], whereas both estimates were higher in comparison to the Irish Women's National League (match, 640.3/1000 h; training, 60.6/1000 h) [23]. The differences identified between the current study, pooled estimates [6], and a study in the Irish Women's National League [23] could be due to the majority of previous studies being conducted over 10 years ago [6]. Also, some of the studies in the review included amateur players [23], and a small number of injuries (range: 20–299 injuries [6]) in comparison to the present study (2167 injuries). Therefore, previous estimates may not reflect that of the current elite and professional game in England, in which higher injury incidence and burden may be a result of many factors including greater training load, higher match intensity, and/or increased reporting of injuries.

Given the larger sample sizes in the current study and the UEFA WECIS [8], this increases confidence in the validity of the estimates of injury incidence and burden associated with modern day elite women's football in Europe. An explanation for the contrasting values reported in the current study compared to the UEFA WECIS, could be a result of numerous contextual factors that can affect the performance of elite football players [24]. Inter‐ and intra‐team differences in styles and standards of play (48% of teams involved in the present study were from the second tier of English women's professional football), infrastructure, resources and fixture/training schedules implemented in different populations may also influence injury characteristics. Now benchmark statistics have been reported in comprehensive datasets, like that of the current study, the cause of different incidences and burdens of injury between women's professional football populations require further exploration to inform future prevention strategies.

### 4.2 Injury Diagnoses, Type, and Location

There are certain injuries that can have a profound impact on individuals' careers and ongoing health, some of which are considered to be of great concern within women's professional football and require extra attention. One such example of this is ACL injuries. It has been well documented that women are at increased risk of sport‐related ACL injuries [25], which is of concern as they often result in surgery, cause prolonged absences from competition [26], and can lead to osteoarthritis in later life [27]. Data on elite women's football in Sweden indicates that ACL injury incidence is 0.14/1000 h [28], which is marginally higher than observed in the current study (0.11/1000 h). Although the difference is slight, the practicality of this small difference remains to be seen. Based on a team reporting 4455 h of exposure per season (median overall exposure of the teams in the present study), this would equate to 0.62 ACL injuries in Sweden compared to 0.49 ACL injuries per team per season in England. The slightly lower total ACL incidence in the present study compared to Sweden may have occurred due to the lower incidence of ACL injuries in match‐play (0.49 vs. 0.72/1000 h) [28]. More recent reports highlight a total, match and training ACL injury incidence of 0.1/1000 h, 0.6/1000 h, and 0.1/1000 h respectively [8]. This indicates that while ACL injuries are still an issue, their occurrence during match‐play is lower than previous research conducted over 10 years ago [28], meanwhile more recent research aligns with the current study [8]. Therefore, it is feasible to suggest that over longer periods of time, improvements in injury prevention strategies, medical provisions, and playing facilities in the women's game perhaps underpin a lower ACL match incidence. Given the present study was able to obtain a large sample size reporting injuries over multiple seasons in an elite population, current estimates are representative of modern women's professional football in England. Interestingly, in comparison to elite men's football, ACL match incidences are comparable to the current study (0.34/1000 h, [29]), although a lower total incidence was observed in men (0.07/1000 h) [29]. A high ACL injury burden was observed by the current study compared to other injuries (32.00 days absent/1000 h, Table 5), as reported in previous research in women's domestic football (38.0 days absent/1000 h [8], 59.00 days absent/1000 h [23]). This particularly applies to match injury burden, which appears higher in the current study compared to previous research (136.31 vs. 117.0, UEFA WECIS) [8]. Moreover, the current study found no significant changes across the study period in match incidence and burden for ACL injury. Overall, the results indicate that ACL injuries occurring in match‐play in women in England appear to be similar to recent reports in elite European football, however, overall incidence remains higher than in elite men. As such, strategies directed toward reducing ACL injuries, and their associated high burden, in elite women's football are still of paramount importance.

The current study identified hamstring injuries as the most common injury diagnosis with an overall incidence of 0.72 injuries/1000 h, which is consistent with recent reports from the UEFA WECIS (0.8/1000 h) [8], and caused the second highest injury burden (18.56 days absent/1000 h). However, these findings are not consistent with those of a recent review, where the most common injury diagnosis in women's football was ankle ligament sprains, followed by quadriceps muscle strains, and knee ligament sprains [6]. In the present study, hamstring injury burden was over 2‐fold higher in comparison to the UEFA WECIS (8.3 days absent/1000 h) [8]. Additionally, the current study found no significant seasonal changes in match and training incidence and burden for hamstring injury. Nevertheless, it is well documented that the demands of women's professional football have increased over time [2, 3], and with varying demands and style of play associated with different domestic competitions, there may be a relationship with differences in the number, severity, and type of injuries that occur in modern women's professional football. The hamstring injury incidence and burden in the current study were lower than the UEFA Elite Club Injury Study (ECIS) in men's professional football (incidence: 4.77; burden: 88.50/1000 h) [30]. Meanwhile, an important observation was that training injury burden of hamstring injuries was higher in comparison to men's football (9.35 vs. 6.30 days absent/1000 h) [30]. Given the substantial developments in modern professional women's football [2, 3], and the need for training to meet the demands of match‐play, it is feasible to suggest the nature of the game could influence the incidence and burden of hamstring injuries. The identification of benchmark statistics within the present study will allow practitioners and stakeholders to consider player welfare in an objective manner.

The present findings show the most common types of injuries in English women's domestic football are muscle and ligament injuries, which is consistent with recent findings in the UEFA WECIS [8]. Due to the dearth of large‐scale epidemiological studies reporting data relating to injury type and location normalized for exposure in elite women's football, comparable data from elite male players may provide useful insights. When compared to the UEFA 18‐year ECIS in men's domestic football [31], ligament injury burden in matches and training are higher in women (match: 266.83 vs. 141.00; training: 24.91 vs. 10.90 days absent/1000 h). However, muscle injury burden in matches is lower compared to men (118.48 vs. 178.40 days absent/1000 h). It has been suggested that there are sex differences in ligament properties including smaller ligament size, decreased femoral notch width, increased joint laxity, and increased body mass index [32, 33, 34], and may be the reason for the sex differences in injury burden. Additionally, training status and increased load particularly in matches, may also impact injury risk in women [4, 5]. This highlights the importance of further sex‐specific research to mitigate ligament injury burden in elite women's football.

Thigh injuries (25.4%), followed by the knee (18.6%), and ankle (18.0%) were the most common location of injury in the present study. This is consistent with previous research in women's football, with studies conducted over the last 20 years also showing the consistent commonality of thigh, knee, and ankle injuries [9, 10, 11, 12, 14, 17, 23]. This suggests that while the demands of women's football have changed over time, injuries at these locations are still common. Although the examination of injury type and location is important to provide useful and comparable information, it is of limited practical utility to medical practitioners when broadly categorized. The ability to identify specific injury diagnoses and analyze incidence and burden statistics is essential for the development of injury epidemiology and is a strength of the current research.

## 5 Limitations

Despite the current study being the most comprehensive assessment of injury epidemiology in women's professional domestic football to date, it is not without limitation. The number of teams per season and the provision of training exposure data varied across the period of the study: between 2018 and 2020, 0%–21% of teams provided training exposure data; between 2020 and 2023 the percentage varied from 50% to 82%. Efforts have been made to increase engagement with clubs through regular communication and webinars to ensure optimal adherence. To achieve large‐scale data collection, data are provided by club medical practitioners. Therefore, some variation in reporting is likely, but specific guidance and follow‐ups were provided to mitigate this. It is also important to note the truncation of the 2019–2020 season enforced by the COVID‐19 pandemic (March 2020), thus the data presented may not be a true representation of a full domestic year. Furthermore, training exposure was calculated by the product of the duration of sessions by the number of players involved. It is acknowledged that the method of recording training exposure did not account for training intensity and format. Therefore, absolute training “load” may have differed between teams. Finally, although all teams competed in the top two tiers of English women's football, it is acknowledged that resources, standard of play, and technical ability can all vary. These differences may have influenced the observed injury characteristics, highlighting the need for future studies to investigate their impact on injury occurrence.

## 6 Perspectives

This study is the largest study of its kind in English women's professional domestic football, conducted over five consecutive seasons, comprising of 93 team seasons, including a total of 2167 injuries across 255 272 h of exposure in matches and training. The main findings show that match injury incidence was 20.5 injuries/1000 h, training injury incidence was 2.9 injuries/1000 h, match injury burden was 775.1 days absent/1000 h, and training injury burden was 96.9 days absent/1000 h. Match injury incidence and burden were greater than training, whereby training injury incidence decreased by 28% across the study period. Hamstring injuries were the most common injury diagnoses, and ACL injury was observed to be the most burdensome in professional women's football. No significant seasonal changes in hamstring and ACL injury incidence and burden were observed which, considering the increase in playing demands, suggests some development to mitigate these injuries. However, prevention strategies to reduce these injuries and their associated burden should continue to be considered by medical practitioners. Importantly, the current study provides benchmark statistics for injury epidemiology in elite English women's domestic football and provides data against which injury prevention strategies can be evaluated.

## Author Contributions

C.C., S.K., P.B. conceptualized the study. Material preparation, data collection, and analysis were performed by all authors. It should be noted that data were collected by the coaches and medical staff caring for each of the relevant teams. C.C., P.B., A.A., and R.K. provided oversight of staff to ensure data were collected appropriately for the injury and illness surveillance project. All authors were involved in the preparation of material used for some data collection, and B.S., P.H., I.V., A.C., S.C., J.M. conducted the analysis. B.S. and P.H. drafted the manuscript. All authors revised the manuscript, contributed to the content, and approved the final version. I.V. is the guarantor.

## Ethics Statement

This study involves human participants, and ethical approval was granted by the Nottingham Trent University Non‐Invasive Ethical Review Committee to retrospectively analyze FA owned data (application number: 116V2).

## Consent

Informed consent was obtained from all clubs prior to data collection.

## Conflicts of Interest

C.C., S.K., P.B., R.K. are or have been employees of the English Football Association. A.A. is an employee of the Women's Professional Leagues Ltd. I.V. has received funding as principal investigator from the English Football Association as an Injury Surveillance consultant. No other conflicts of interest to report.

## Data Availability

The data are not publicly available due to their containing information that could compromise the confidentiality of clubs and organizations involved.
